# Adherens junctions connect stress fibres between adjacent endothelial cells

**DOI:** 10.1186/1741-7007-8-11

**Published:** 2010-02-02

**Authors:** Jaime Millán, Robert J Cain, Natalia Reglero-Real, Carolina Bigarella, Beatriz Marcos-Ramiro, Laura Fernández-Martín, Isabel Correas, Anne J Ridley

**Affiliations:** 1 University College London, Ludwig Institute for Cancer Research and Department of Biochemistry and Molecular Biology, London WC1E 6BT, UK; 2 Centro de Biología Molecular Severo Ochoa, CSIC-Universidad Autónoma de Madrid, Cantoblanco, 28049 Madrid, Spain; 3 Randall Division of Cell and Molecular Biophysics, King's College London, New Hunt's House, Guy's Campus, London SE1 1UL, UK

## Abstract

**Background:**

Endothelial cell-cell junctions maintain endothelial integrity and regulate vascular morphogenesis and homeostasis. Cell-cell junctions are usually depicted with a linear morphology along the boundaries between adjacent cells and in contact with cortical F-actin. However, in the endothelium, cell-cell junctions are highly dynamic and morphologically heterogeneous.

**Results:**

We report that endothelial cell-cell junctions can attach to the ends of stress fibres instead of to cortical F-actin, forming structures that we name discontinuous adherens junctions (AJ). Discontinuous AJ are highly dynamic and are increased in response to tumour necrosis factor (TNF)-α, correlating with the appearance of stress fibres. We show that vascular endothelial (VE)-cadherin/β-catenin/α-catenin complexes in discontinuous AJ are linked to stress fibres. Moreover, discontinuous AJ connect stress fibres from adjacent cells independently of focal adhesions, of which there are very few in confluent endothelial cells, even in TNF-α-stimulated cells. RNAi-mediated knockdown of VE-cadherin, but not zonula occludens-1, reduces the linkage of stress fibres to cell-cell junctions, increases focal adhesions, and dramatically alters the distribution of these actin cables in confluent endothelial cells.

**Conclusions:**

Our results indicate that stress fibres from neighbouring cells are physically connected through discontinuous AJ, and that stress fibres can be stabilized by AJ-associated multi-protein complexes distinct from focal adhesions.

## Background

Endothelial cell-cell junctions maintain endothelial integrity and regulate vascular morphogenesis. A major role of the vascular endothelium is to control the movement of small solutes and leukocytes in and out of the bloodstream. Endothelial junctions consist of several different multi-protein complexes, whose relative abundance and roles in regulating permeability and leukocyte diapedesis depend on the endothelial cell type. Endothelial adherens junctions (AJ) and tight junctions (TJ) are the main regulators of paracellular permeability in the endothelium. Some junctional proteins unique to endothelial cells, including PECAM-1, ICAM-2 and S-endo I, also contribute to endothelial barrier function [1]. In endothelial AJ, the transmembrane vascular endothelial (VE)-cadherin binds the cytoplasmic proteins β-catenin and p120-catenin. β-catenin also binds α-catenin, which could link the AJ complex to actin filaments [2]. However, the established model of a direct link between cortical actin filaments (F-actin) and α-catenin in AJ in epithelial cells has been questioned by data demonstrating that the binding of α-catenin to β-catenin or F-actin is mutually exclusive, and suggesting that α-catenin stabilizes AJ by regulating actin polymerization instead of by linking F- actin to AJ [3,4].

Endothelial cell-cell junctions are regulated by a variety of extracellular stimuli, which often act by inducing reorganization of the actin cytoskeleton. For example, Rho guanosine triphosphate (GTPases) and their targets, the rho serine/threonine kinases (ROCKs), stimulate actomyosin-based contractility, generating stress fibres and focal adhesion (FA) and thereby contribute to the rapid increase in endothelial permeability in response to thrombin and histamine [5-8]. Stress fibres generated in response to these stimuli also reorganize junctional complexes [9-11]. Pro-inflammatory stimuli such as tumour necrosis factor (TNF)-α also induce long-term changes to endothelial cell-cell junctions, actin stress fibre reorganization and an increase in permeability [12,13].

Cell-cell junctions are usually depicted with a linear morphology along the boundaries between adjacent cells and in contact with cortical F-actin. Here we describe the distinct properties of endothelial cell-cell junctions that localize at the ends of stress fibres that we name discontinuous AJ. These structures are distinct from focal adhesions, which are found at the ends of stress fibres in subconfluent endothelial cells. In response to TNF-α, association of stress fibres with discontinuous AJ, but not focal adhesions, is increased, suggesting that AJ may play a role stabilizing stress fibres in confluent endothelial cells.

## Results

### Composition and dynamics of discontinuous AJ

**Figure 1:**
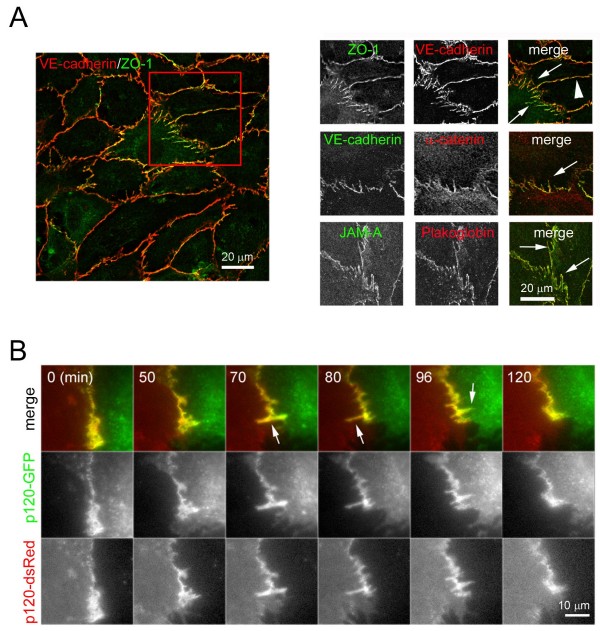
**Composition of discontinuous adherens junction (AJ)**. (A) Human umbilical vein endothelial cells (HUVECs) were grown at confluency for 72 h in EGM-2 growth medium, fixed, permeabilized and stained with antibodies to the indicated junctional proteins. Top right panels show single staining of the merged image on the left. Arrows indicate discontinuous cell-cell junctions; arrowhead, linear cell-cell junctions. (B) HUVECs were nucleofected with plasmids coding for p120-catenin-green fluorescent protein (p120-GFP) or p120-catenin-red fluorescent protein (p120-dsRed), cells from the two transfections were mixed and plated at confluence for 24 h in growth medium. Cell images were acquired by fluorescence time-lapse microscopy, 1 frame/min. Representative images are shown. Arrows show discontinuous AJ.

Analysis of cell-cell junction distribution in human umbilical vein endothelial cells (HUVECs) revealed that, as well as localizing linearly along cell-cell borders similar to junctions in epithelial cells (Figure 1A, arrowhead), in some areas junctional proteins were distributed in multiple short linear structures that were almost orthogonal to cell-cell borders (Figure 1A, arrows) [14]. AJ components such as VE-cadherin, α-catenin, β-catenin or plakoglobin, TJ components such as zonula occludens-1 (ZO-1) and other junctional proteins, such as JAM-A or CD99 (not shown), appeared in these short linear structures that were often clustered in regions along cell-cell borders (Figure 1A). In some cases, these structures branched off linear junctional regions (Figure 1A, arrows). We call these structures discontinuous junctions, because the linear distribution along cell-cell borders is broken in these areas. In order to analyse the dynamics of discontinuous junctions, cells transiently expressing the AJ component p120-catenin tagged either with green fluorescent protein (p120-GFP) or red fluorescent protein (p120-dsRed) were mixed and analysed by time-lapse fluorescence microscopy (Figure 1B, Additional File 1). Endothelial AJ were highly dynamic and reorganized continually. Discontinuous junction linear structures orthogonal to the cell-cell border appeared and disappeared rapidly, often within a few minutes (Figure 1B. arrows, Additional File 1). Discontinuous junctions were generally formed from both adjacent cells, with an extension from one cell overlaying the structure within the neighbouring cell (Figure 1B, arrows, Additional File 1). Their appearance usually coincided with retraction of one cell border with respect to the other, leaving a finger-like protrusion and their disappearance occurred when one cell border extended back over the protrusion area. These results indicate that dynamic behaviour of endothelial cell-cell borders is responsible for the formation of discontinuous AJ structures.

### Discontinuous AJ are associated with stress fibres at cell-cell borders

**Figure 2:**
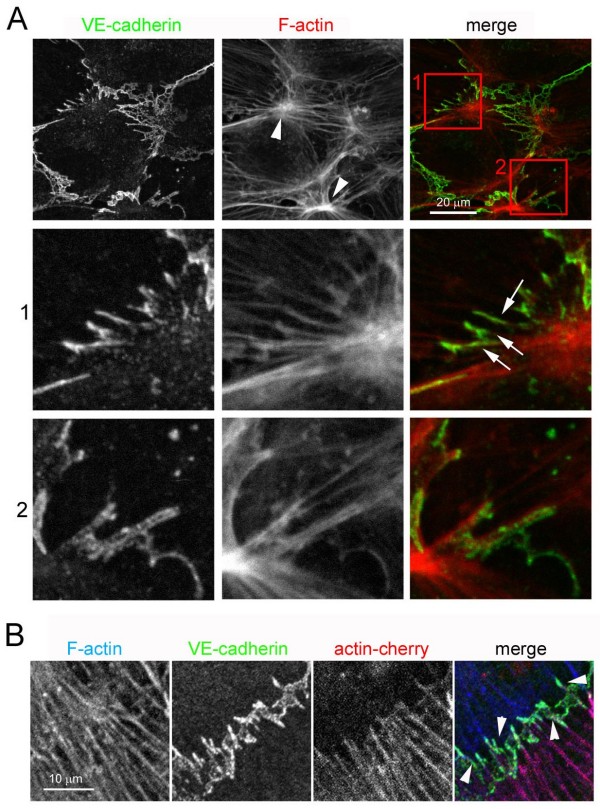
**Discontinuous adherens junction (AJ) anchor stress fibres at cell borders**. (A) Confluent human umbilical vein endothelial cells (HUVECs) in EGM-2 growth medium were stained for actin filaments (F-actin; TRITC-phalloidin) and vascular endothelial (VE)-cadherin. Bottom panels are enlargements of red boxed areas from top panel that are enriched in discontinuous AJ. (B) HUVECs were nucleofected with a plasmid encoding actin-cherry and plated at confluence for 24 h to 48 h in growth medium. Cells were fixed and stained for VE-cadherin or F-actin. Arrowheads indicate discontinuous AJ.

Cell-cell junctions are regulated by cortical F-actin [5]. In discontinuous AJ, VE-cadherin was found to align with the ends of stress fibres, some of which traversed the cell body form one side to the other. Stress fibres in one cell were often connected to stress fibres of adjacent cells through discontinuous AJ (Figure 2A, arrows) and discontinuous AJ structures clustered in regions where stress fibres coalesced to form stellate arrangements (Figure 2A, arrowheads). Analysis of adjacent cells where one cell expressed actin-Cherry and the other did not confirmed that discontinuous AJ can connect stress fibres between neighbouring cells (Figure 2B, arrowheads), suggesting that AJ complexes anchor stress fibres at endothelial cell-cell borders.

**Figure 3:**
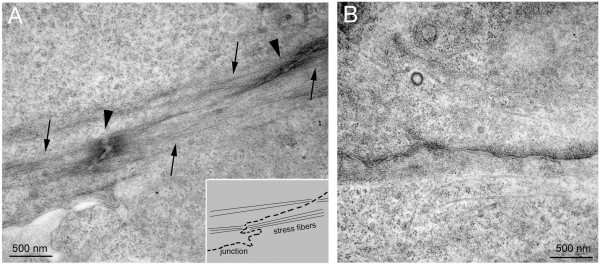
**Electron microscopy analysis of endothelial cell-cell borders**. Confluent human umbilical vein endothelial cells (HUVECs) either (B) unstimulated or (A) tumour necrosis factor-α-stimulated for 18 h were fixed and processed for electron microscopy. Arrows denote actin filament bundles, whilst arrowheads indicate position of electron-dense junctional structures. Inset: cartoon of panel A illustrating placement of stress fibres with respect to junctional contacts, confirming a connection between junctions and actin filaments, which is not observed at linear junctions (B).

Electron microscopy analysis of endothelial cell-cell borders confirmed the connection of F-actin bundles (Figure 3A, arrows), distinct from subcortical F-actin, from neighbouring cells through electron-dense junctional structures (Fig. 3A, arrowheads). The shape of the intercellular junctional perimeter in relation to such cables was suggestive of tension exerted by these fibres on the connected junctions (Figure 3A, inset). In contrast, linear junctional regions showed no associated actin cables (Figure 3B).

### Discontinuous AJ are associated with the end of stress fibres independently of focal adhesions (FA)

**Figure 4:**
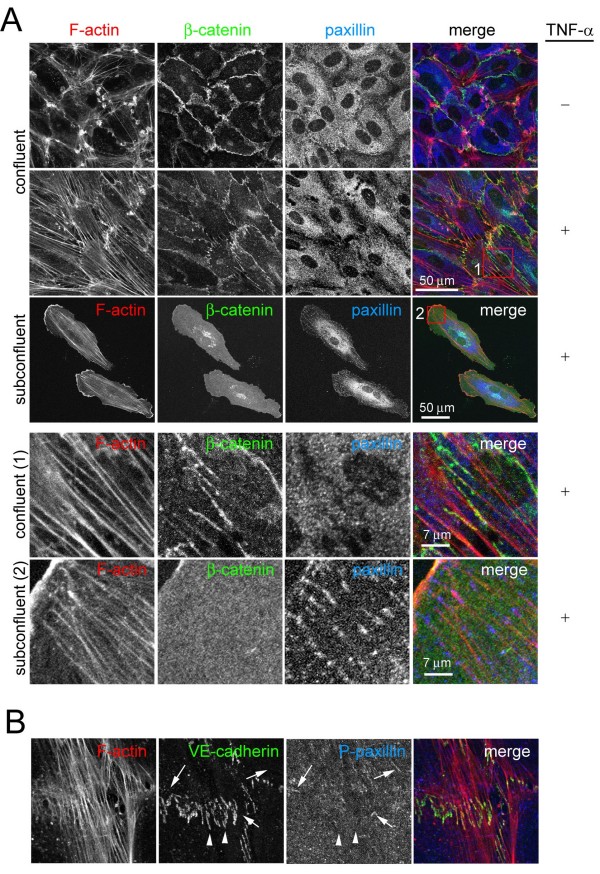
**Effect of tumour necrosis factor (TNF)-α and confluence on discontinuous adherens junction (AJ), stress fibres and focal adhesions**. (A) Effect of TNF-α on actin filament, β-catenin and paxillin distribution. Confluent human umbilical vein endothelial cells (HUVECs) were starved for 4 h and then, as indicated, stimulated with 10 ng/ml TNF-α for 20 h. In parallel, subconfluent HUVECs were also starved and stimulated with TNF-α. Cells were fixed and distribution of F-actin, β-catenin and paxillin was analyzed by immunofluorescence. Bottom panels show the red boxed areas indicated in merged images at higher magnification. (B) Distribution of F-actin, VE-cadherin and phospho-Y(118)-paxillin in TNF-α-stimulated confluent HUVECs. Arrowheads indicate discontinuous AJ, whereas arrows indicate focal adhesions detected by phospho-paxillin staining.

**Figure 5:**
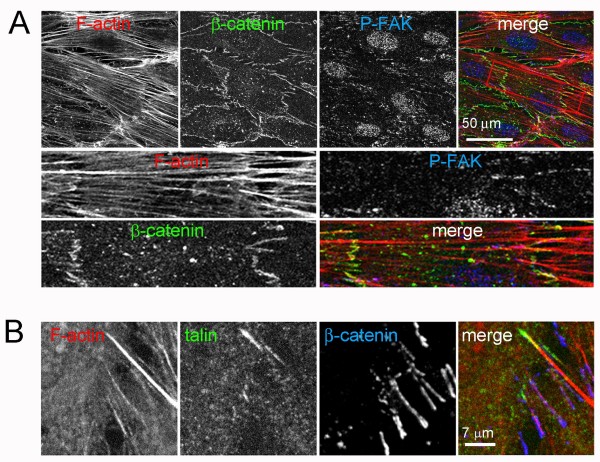
**Discontinuous adherens junctions are distinct from focal adhesions**. Confluent human umbilical vein endothelial cells were stimulated with 10 ng/ml tumour necrosis factor-α as in Figure 4. Cells were fixed and stained with TRITC-phalloidin, in order to detect actin filaments, with antibodies to β-catenin, phosphorylated FAK (p(Y397)FAK) (A) or TRITC-phalloidin and antibodies to detect talin and β-catenin (B). Bottom panels in (A) show the red-boxed area from the top panels (merge) at higher magnification.

**Figure 6:**
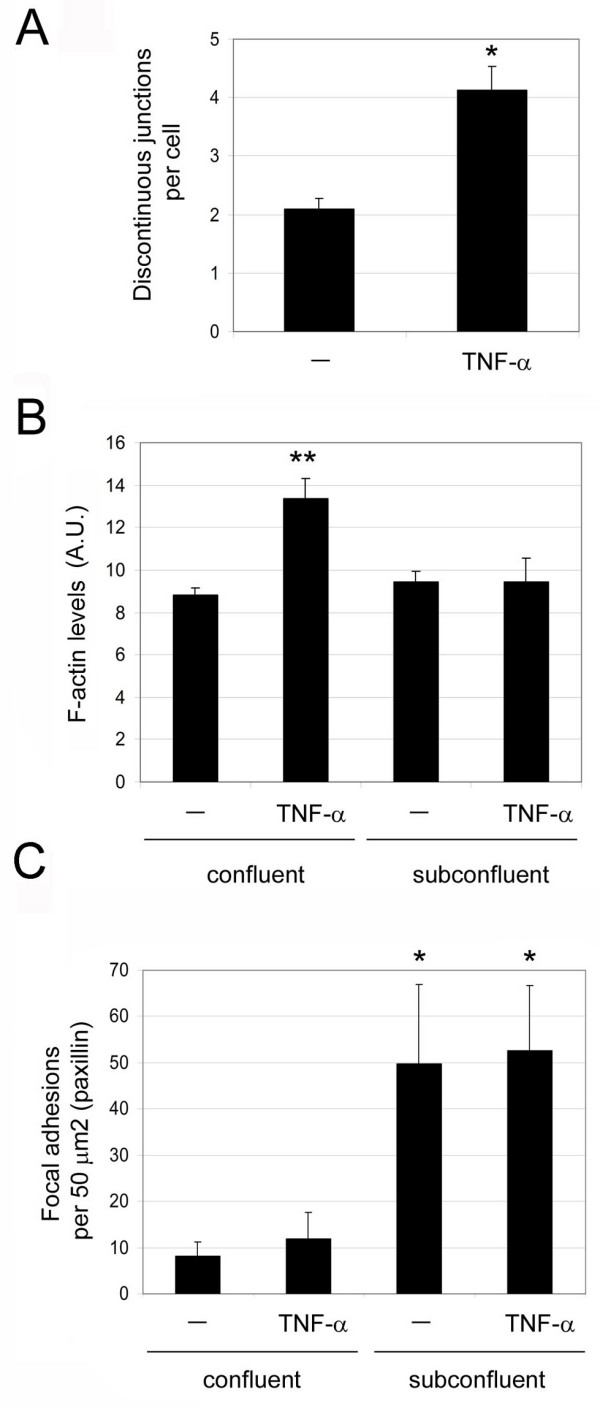
**Quantitation of the effect of tumour necrosis factor (TNF)-α on discontinuous adherens factor, stress fibres and focal adhesions (FAs) in confluent and subconfluent human umbilical vein endothelial cells (HUVECs)**. (A) Quantitation of discontinuous junctions per cell with or without TNF-α stimulation. (B) Quantitation of actin filament staining from confocal images between confluent and subconfluent HUVECs with and without TNF-α stimulation as in Figure 4. (C) Quantitation of FA-like paxillin clusters in confluent and subconfluent HUVECs with and without TNF-α stimulation. Error bars indicate standard error of mean; *n *≥ 3 experiments. *, *P *< 0.008; **, *P *< 0.001, compared to confluent unstimulated cells. AU, arbitrary units.

In most cultured cells, stress fibres are linked to FA, where integrins cluster [15]. Pro-inflammatory stimuli such as TNF-α or IL-1β induce actin stress fibres, cell elongation and contractility in endothelial cells [12] (Figure 4A). We therefore hypothesized that pro-inflammatory stimuli would alter the relative levels of discontinuous AJ. Endothelial cells were grown at confluency for 3 days and stimulated with TNF-α for 20 h. TNF-α induced cell elongation and an increase in stress fibres that were aligned parallel with the elongated axis of the cells, as previously described [13]. These stress fibres often appeared attached to discontinuous AJ, which were also increased in response to TNF-α (Figures 4, 5 and 6A). In these regions, stress fibres were frequently aligned in neighbouring cells (Figure 4B and 5A, boxed area). The TNF-α mediated induction of stress fibres (Figure 4A) was accompanied by an overall increase of F-actin detected by quantitation of phalloidin staining (Figure 6B). However, these stress fibres were rarely attached to FA as identified by localization of paxillin, a FA protein [16] (Figure 4A) and TNF-α did not induce a significant increase in the number of FA (Figure 6C). In contrast, stress fibres in sub-confluent cells were found attached to FA, which were far more abundant in sub-confluent compared to confluent cells (Figure 4A and 6C), even though the F-actin content and stress fibre levels of confluent and subconfluent HUVECs was comparable (Figures 4A and 6B), and even higher in response to TNF-α at confluence. This is in clear contrast to previously described responses to acute inflammatory stimuli, such as thrombin, which induce the formation of FAs concomitant with an increase in stress fibres (data not shown and [7,17-19]). Interestingly, TNF-α did not stimulate any detectable change in F-actin in subconfluent cells (Figure 6B). Finally, discontinuous AJ attached to stress fibres were not found associated with FA, since they did not co-localize with the FA components phospho(pY118)-paxillin (Figure 4B), talin or phospho(Y397)-focal adhesion kinase (FAK) (Figure 5), indicating that FA and discontinuous AJ are separate structures that anchor stress fibres in different areas of the endothelial monolayer [16].

**Figure 7:**
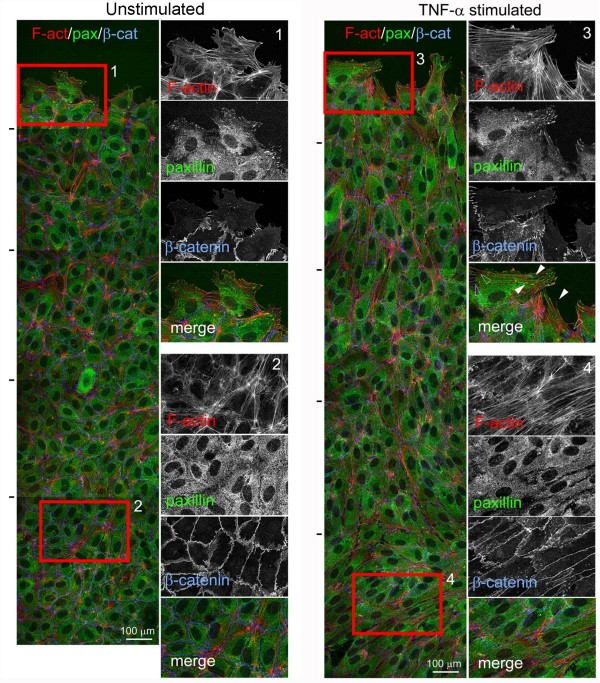
**Effect of tumour necrosis factor (TNF)-α and confluence on discontinuous adherens junctions, stress fibres and focal adhesions**. Confluent unstimulated human umbilical vein endothelial cells (HUVECs) (A), or HUVECs stimulated with 10 ng/ml TNF-α for 20 h (B) in growth medium, were scratched with a plastic tip and after 5 h cells were fixed and stained for actin filament, paxillin and β-catenin. All images are projections of *z*-stacks of confocal images. Left images show a general view of five confocal fields sequentially acquired and superimposed from the scratch edge (top) into the confluent monolayer (bottom). Black lines on left indicate the edges of each confocal field. Right panels show the boxed areas at higher magnification, either at the scratch edge (1 and 3) or within the confluent monolayer (2 and 4). Arrowheads show stress fibre tips associated to paxillin clusters, empty arrowheads points to the tips of the same stress fibres associated to discontinuous junctions on the other side.

**Figure 8:**
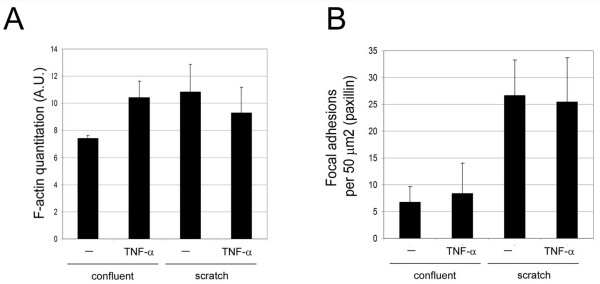
**Quantitation of the effect of tumour necrosis factor (TNF)-α and confluence on stress fibres and focal adhesions (FAs) during the scratch asssay**. Quantitation of actin filament levels (A) and FA-like paxillin clusters (B) from confocal images of cells at the scratch edge (scratch) and cells in confluent areas (confluent) with and without TNF-α. AU, arbitrary units.

In order to analyse in more detail the difference in stress fibre attachment sites between cells with and without cell-cell adhesions, we compared cells at the edge of scratch wounds with cells far from the scratch. Cells situated at the border of the wound had numerous stress fibres linked to FAs, whereas cells situated in the confluent region of the same monolayer had far fewer FA although stress fibre levels and F-actin were comparable (Figure 7; quantitation in Figure 8). Moreover, some stress fibres in cells at the scratch wound edge appeared connected to FA at one side and discontinuous junctions at the other (Figure 7, arrowheads) Altogether, these data suggest that discontinuous AJ can anchor the ends of stress fibres independently of FA in confluent endothelial cells.

### Stress fibre tension mediates formation of discontinuous junctions

**Figure 9:**
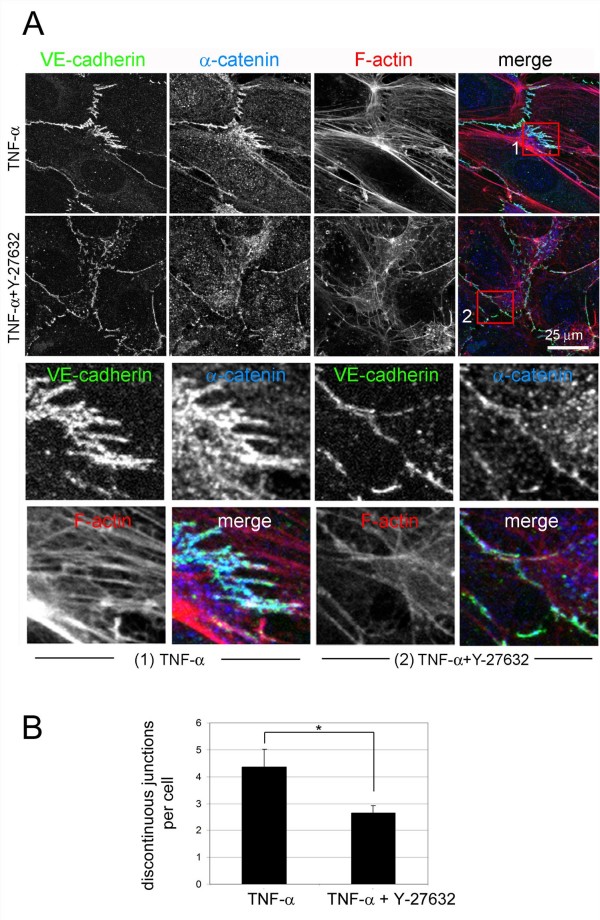
**ROCK inhibition induces loss of discontinuous adherens junction**. Human umbilical vein endothelial cells were plated at confluence for 48 h and stimulated, as indicated, with 10 ng/ml tumour necrosis factor (TNF)-α in growth medium or TNF-α with 5 μM Y-27632. Cells were then fixed and stained for vascular endothelial (VE)-cadherin, α-catenin and actin filament. Bottom panels show the boxed areas indicated in the merge images at higher magnification: (1) TNF-α, (2) TNF-α + Y-27632. (B) Quantitation of discontinuous junctions in TNF-α-stimulated cells with or without 5 μM Y-27632, +/- standard error of mean of three different experiments. *, *P *< 0.016.

Our data indicate that discontinuous AJ form due to dynamic movements of cell-cell borders of adjacent endothelial cells and that they are linked to stress fibres. To investigate whether stress fibres directly affect the formation of discontinuous AJ, cells were treated with the ROCK inhibitor Y-27632, which significantly reduced stress fibres in TNF-α-stimulated HUVECs (Figure 9). Y-27632 reduced the number of discontinuous AJ, although the overall level of junctional VE-cadherin did not appear to be affected and instead the majority of AJ proteins had a linear distribution along cell-cell borders. This indicates that discontinuous AJ are formed as a consequence of stress fibre-induced tension acting on cell-cell junctions. Strikingly, ROCK inhibition did not prevent the permeability increase caused by TNF-α, indicating that this cytokine induces long-term changes in endothelial junctional composition that regulate permeability, independent of stress fibre assembly (data not shown and [13]).

### AJs are necessary for stress fibre attachment to cell-cell junctions

**Figure 10:**
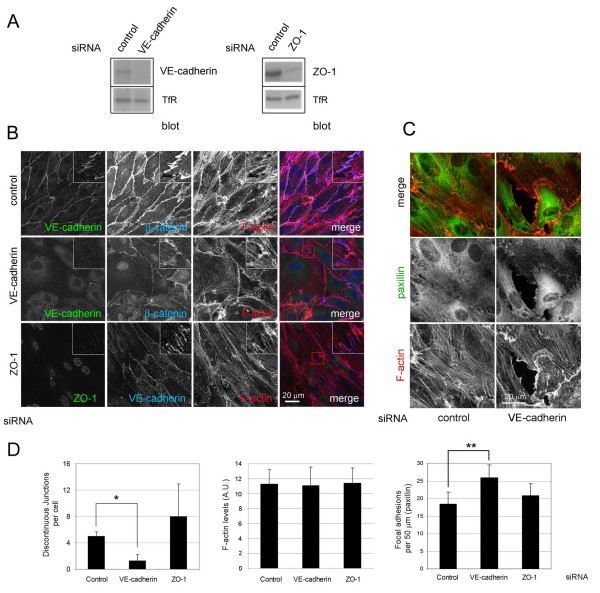
**Vascular endothelial (VE)-cadherin and zonula occludens (ZO)-1 in junctional actin organization**. Human umbilical vein endothelial cells (HUVECs) were transfected with small interfering RNA (siRNA) oligonucleotides targeting VE-cadherin, ZO-1 or a non-specific oligonucleotide control. After 24 h cells were trypsinized and plated at confluence and analysed 48 h later (72 h after transfection). Twenty hours before the analysis cells were stimulated with tumour necrosis factor (TNF)-α in growth medium. (A) Cells were lysed and the effect of each siRNA on the levels of VE-cadherin, ZO-1 and transferrin receptor (TfR) were analysed by western blotting. (B, C) siRNA-transfected HUVECs were stimulated with TNF-α, fixed and stained for the indicated junctional proteins and actin filament (F-actin) (B) or F-actin and paxillin (C). (D) Quantitation of discontinuous junctions, F-actin content and focal adhesions in siRNA-treated cells upon TNF-α stimulation. * *P *< 0.045; ** *P *< 0.065.

In order to determine the contributions of cell-cell junctional components to stress fibre attachment to the junctions, VE-cadherin and ZO-1, components of AJ and TJ, respectively, were knocked down with short interfering RNAs (Figure 10A). VE-cadherin depletion did not completely inhibit the association of β-catenin with cell-cell borders, probably reflecting an association of β-catenin with other cadherins or other junctional components such as PECAM-1 [20,21]. However, this remaining β-catenin no longer showed any localization to discontinuous structures in TNF-α-stimulated cells (Figure 10B and 10D). VE-cadherin depletion did not alter the overall F-actin content (Figure 10D) but altered the distribution of F-actin filaments: there was a decrease in parallel bundles of stress fibres attached to the junctions and, instead, stress fibres were often oriented in multiple directions and many terminated in FAs (Figure 10C). Cortical F-actin increased and cells no longer had an elongated shape (Figure 10B). In contrast, knockdown of ZO-1 did not significantly affect the distribution of AJ and actin stress fibres or cell elongation, suggesting that TJ are not so important for the maintenance of these structures.

## Discussion

We show here that confluent HUVECs contain significantly less FA than subconfluent cells, even after stimulation with TNF-α which induces a large increase in stress fibres and overall F-actin content in confluent cells. Stress fibre formation is due to actomyosin contractility which requires them to be attached somewhere at both ends. Here we demonstrate that stress fibres are attached to VE-cadherin-mediated junctional complexes, although it is likely that some other junctional proteins are also linked to endothelial stress fibres, such as junctional adhesion molecule (JAM)-A or ZO-2-associated tight junctions. VE-cadherin engagement in confluent endothelial cells has been shown to reduce FA by modulating cell tension and spreading via RhoA [22]. In accordance with this, VE-cadherin reduction by small interfering RNA (siRNA) significantly increased FA in confluent cells, although it did not alter the F-actin content. These results explain previous observations on the distribution of tyrosine phosphorylated proteins in endothelial cells which, in subconfluent cells, localized predominantly in a FA-like pattern whereas in confluent cells they were mostly at intercellular borders [23]. The dynamic inter-conversion between linear and discontinuous AJ suggests that there could be a rapid tension-regulated switch of AJ proteins, which may be linked either to F-actin structures that protect the endothelial barrier, such as cortical F-actin [24], or to stress fibres. This link might be regulated, for example, by tension-induced unfolding of a linker protein in AJ, as has been postulated for p130Cas in FA [25].

Interestingly, cadherin complexes have been reported to associate with F-actin similar to stress fibres in transformed epithelial cells and endothelial cells undergoing remodelling (for example in response to wound healing or acute pro-inflammatory stimuli) [9-11,26]. In epithelial cells, E-cadherin has also been shown not to associate with F-actin via α-catenin. It has been proposed that there is no direct association between AJ and the cortical F-actin [4], although it remains possible that another AJ protein can connect AJ to cortical F-actin. Here, based on the specific distribution of discontinuous AJ, together with an analysis of the dynamics of the AJ component p120-catenin and F-actin and the effect of VE-cadherin depletion on stress fibres, we propose that endothelial discontinuous AJ formed by complexes of VE-cadherin, α-catenin, β-catenin and p120-catenin, can be physically linked to actin stress fibres. It is possible that a tension-regulated AJ protein links AJ to stress fibres only under tension but not in resting conditions where linear endothelial AJ are more similar to epithelial AJ and co-localize with cortical F-actin [26]. In agreement with this, stimuli that enhance endothelial barrier properties, such as cyclic adenosine monophosphate, increase the cortical F-actin belt but decrease stress fibres [27]. Taken together, our results and those of others indicate that, both in endothelial and epithelial cells, the association of F-actins such as stress fibres with AJ is regulated and not constitutive. This regulated association would, for example, facilitate cell-cell junction remodelling during wound healing or in response to inflammatory stimuli.

Another role of endothelial stress fibres during inflammation is likely to be the regulation of leucocyte transmigration. Adhesion receptors involved in leucocyte adhesion, such as ICAM-1 or VCAM-1 and transmigration, align with stress fibres in response to engagement [28,29]. ICAM-1 engagement increases RhoA activity and stress fibres [28] as well as regulating phosphorylation of VE-cadherin [30,31]. ICAM-1 and VCAM-1 crosslinking alter the integrity of VE-cadherin cell contacts [31,32]. In the light of these previous results, our data suggest that stress fibres are connectors from apically localized receptors to cell-cell junctions, which may contribute to leukocyte transmigration during inflammation. Stress fibres thus not only regulate endothelial permeability to small solutes, but may also help to withstand the mechanical stress generated by leukocyte transmigration under shear stress. We propose that the linkage of stress fibres between neighbouring cells via discontinuous AJ contributes to increase stress resistance and to regulate the whole endothelial monolayer response to inflammation.

## Conclusions

The current model for cell-cell junctional organization is largely inspired by studies in epithelium. In epithelial cells AJ and TJ are separately organized and associated with cortical actin, although recently it has been proposed that AJ are not directly linked to cortical actin [4]. The endothelium requires more dynamic and heterogeneous cell junctions in order to coordinate fast and local permeability increases to small molecules and cells from the bloodstream. Here we have shown that the ends of actin stress fibres, key actors in leukocyte transmigration and paracellular permeability regulation, are associated with cell-cell junctions. Cell-cell junctions can even connect stress fibres from neighbouring cells. Finally, we provide clear evidence that AJ are supra-molecular protein complexes distinct to FAs able to stabilize stress fibres in confluent endothelial cells. Our results clearly show a distinct organization of endothelial F-actin at confluence which is likely to be relevant during permeability changes and leukocyte transendothelial migration and for endothelial inflammatory migration and angiogenesis.

## Methods

### Antibodies

Mouse monoclonal anti-VE-cadherin blocking antibody was obtained from BD Pharmingen (CA, USA; Cat. 555661). Anti-VE-cadherin (Cat. 610252), anti-α-catenin, anti p120-catenin, anti-FAK, anti-phospho-Y397-FAK and anti-paxillin (610051) mouse monoclonal antibodies were obtained from BD Transduction Laboratories (NJ, USA). Anti-β-catenin (C-2206) rabbit polyclonal and anti-talin (T-3287) mouse monoclonal antibodies were obtained from Sigma (Melbourne, Australia). Anti-α-catenin rabbit polyclonal antibody was obtained from Santa Cruz (CA, USA). Anti-γ-catenin mouse monoclonal antibody, anti-ZO-1 and anti-JAM-A rabbit polyclonal antibodies were obtained from Zymed (CA, USA). Anti-pY118-paxillin phospho-specific antibody (44-722) was obtained from Biosource (Nivelles, Belgium).

### Cell culture and transfection

HUVECs were obtained from Lonza (Wokingham, UK). They were cultured in Nunclon flasks pre-coated with 10 μg/ml human fibronectin in endothelial basal medium (EBM-2; (Lonza, MD, USA) supplemented with 2% fetal bovine serum (FBS), endothelial cell growth supplement EGM-2 (Lonza) (growth medium) in an atmosphere of 5% CO_2_/95% air. When experiments were performed in starving conditions, confluent HUVECs were starved in EBM-2 medium supplemented with 1% fetal calf serum (starvation medium) prior to stimulation with 10 ng ml^-1 ^TNFα. No major differences in FA content were detected in cells stimulated in growth medium or starving medium.

HUVECs were transiently transfected with 1-5 μg plasmid DNA/10^6 ^cells with a Nucleofector kit (VPB-1002) (Amaxa Biosystems, Cologne, Germany) according to the manufacturer's instructions, and used for experiments 24 h to 72 h after transfection. For experiments with cells expressing different fluorescently-tagged proteins in the same monolayer, 3 × 10^6 ^cells were transfected with each plasmid, pooled together, plated on two glass-bottom 35-mm dishes (MatTek Corporation, MA, USA) or two glass coverslips previously coated with fibronectin for 15 h and analysed 24 to 48 h after transfection. Since nucleofection can induce significant cell death, once transfected cells were plated, untransfected cells were sometimes added in order to provide sufficient cells to form of a confluent monolayer.

For siRNA transfection, a protocol derived from a modification of our previous method [29] was used for delivery of siRNA with high efficiency into primary endothelial cells. HUVECs were plated at sub-confluence (10^5 ^cells on each well of a six-well dish) in EBM-2 medium with no antibiotics. The following day cells were transfected by mixing 4 μl of oligofectamine with siRNA to a final concentration of 100 nM. Twenty-four hours after transfection cells were trypsinized and plated at confluence onto different dishes in order to perform parallel assays such as immunofluorescence and western blotting. Assays were performed 72 h after transfection.

### Plasmids and siRNAs

In order to construct the p120-DsRed plasmid, p-EGFP-120^ctn^- (generous gift from Keith Burridge) and dsRed vector from Clontech (CA, USA) were sequentially digested with AgeI and NotI enzymes and, then, the p120 vector without the GFP and the DsRed were ligated using the Ligase enzyme (New England Biolabs, Massachusetts, USA). β-Actin-GFP was a generous gift from Dr Beat Imhof. β-actin-Cherry was a generous gift from Ke Hu and Dr Ann Wheeler. The following siRNA oligonucleotides were obtained from the predesigned siGenome collection of Dharmacon (IL, USA). D-003641-01 (VE-cadherin), D-007746-01 (ZO-1). D-001210-01 (control (1)) or D-001810-01 (control (2)) non-targeting siRNA were used as controls in different experiments.

### Confocal and time-lapse microscopy

For confocal microscopy, cells were fixed with 4% paraformaldehyde for 20 min, or at -20°C in 100% methanol for 5 min, for the detection of Talin. They were then blocked with tris-buffered saline (25 mM Tris pH 7.4, 150 mM NaCl) for 10 min, permeabilized for 5 min with phosphate buffered saline (PBS) containing 0.2% Triton X-100 at 4°C, blocked with PBS containing 1% bovine serum albumin and incubated at 37°C with primary then fluorophore-conjugated secondary antibodies or 1 μg/ml TRITC/FITC-labelled phalloidin. Specimens were mounted in DAKO fluorescent mounting medium (DAKO Corporation, CA, USA).

Confocal laser scanning microscopy was carried out with an LSM 510 (Zeiss, Welwyn Garden City, UK) mounted over an Axioplan microscope (Zeiss) using a ×40 1.3 NA oil immersion objective. In order to obtain Z stacks, three to six optical sections were taken over 4 μm. Intensity profiles were generated using the Zeiss LSM software.

Time-lapse microscopy was performed with a Nikon TE2000-E Eclipse Inverted microscope, in an environmental chamber at 37°C. 20 mM HEPES (pH 7.4) was added to the cells 2 h before the experiment. Images were taken with a cooled CCD camera Hamamatsu Orca-ER C4742-95. Camera and shutter (Lambda Instruments, VA, USA) were controlled by Andor Q software. Movies were processed with Metamorph software.

### Electron microscopy

TNF-α-stimulated or unstimulated HUVECs were fixed for electron microscopy using 2% glutaraldehyde in 60 mM PIPES, 25 mM HEPES, pH 7.3, 3 mM MgCl_2_, 10 mM EGTA and 1% Triton TX-100, treated with 1% osmium tetroxide and dehydrated through a graded series of ethanol. The samples were then embedded in TAAB resin by conventional procedures, and 70 nm sections were cut using a Leica Ultracut E ultra microtome (Leica, Vienna, Austria). Sections were mounted onto 200 mesh copper grids and stained with lead citrate, before viewed on a H7600N transmission electron microscope (Leica). Digital images were captured using an AMT camera (Deben, Suffolk, UK).

### Quantification of F-actin, FAs and discontinuous junctions

Confocal images from 8 to 30 cells per experiment from at least three different experiments were contrasted and analysed using LSM 510 software (Zeiss) in order to distinguish morphologically discontinuous junctions or FA-like paxillin staining at the basal planes. For F-actin quantification, images from cells stained for TRITC-labelled phalloidin were exported in formats compatible with ImageJ software. ImageJ was used to obtain the mean fluorescent intensities from different cellular regions of confluent cells, subconfluent cells or cells at the border of a wound induced in a confluent monolayer with a plastic tip 5 h before. Data were processed and statistical significance determined using Student's *t*-test (Microsoft Excel).

## Abbreviations

AJ: adherens junctions; cAMP: cyclic adenosine monophosphate; DsRED: red fluoroescent protein; FA: focal adhesion; F-actin: actin filament; GFP: green fluorescent protein; HUVEC: human umbilical vein endothelial cells; PBS: phosphate buffered saline; ROCK: rho kinase; siRNA: small interfering RNA; TJ: tight junctions; TNF: tumour necrosis factor; VE: vascular endothelial; ZO1: zonula occludens-1; FAK: focal adhesion kinase.

## Authors' contributions

JM designed experiments and performed experiments and wrote the manuscript. RC performed the electron microscopy experiments. NR did some of the immuno localizations of cell-cell junctional proteins. BM did some immunolocalizations and subsequent quantitations, LF maintained cell cultures, transfected plasmids and siRNAs and prepared cells on coverslips. IC provided cells, antibodies specific for junctional proteins and corrected the manuscript. CB started the project and made the p120-catenin-dsRed cDNA construct. AR supervised the project, designed experiments and wrote the manuscript.

## Supplementary Material

Additional file 1 **Movie 1**. Human umbilical vein endothelial cells were nucleofected with plasmids encoding p120-catenin-green fluorescent protein and p120-catenin-red fluorescent protein (p120-dsRed) and plated at confluence for 24 h in growth medium. Cell images were acquired by fluorescence time-lapse microscopy, 1 frame/min. (See Figure 1.) Click here for file
